# ﻿Further investigation of *Melocalamus* (Poaceae, Bambusoideae) in China based on Skmer analysis and morphology

**DOI:** 10.3897/phytokeys.259.151683

**Published:** 2025-07-02

**Authors:** Yu-Jin Chen, Mei Chen, Meng-Yuan Zhou, Zu-Chang Xu, Yu-Xiao Zhang, Jing-Xia Liu, De-Zhu Li

**Affiliations:** 1 Germplasm Bank of Wild Species & Yunnan Key Laboratory of Crop Wild Relatives Omics, Kunming Institute of Botany, Chinese Academy of Sciences, Kunming, Yunnan 650201, China Chinese Academy of Sciences Kunming China; 2 University of Chinese Academy of Sciences, Beijing 100049, China University of Chinese Academy of Sciences Beijing China; 3 Institute of Biodiversity, School of Ecology and Environmental Science, Yunnan University, Kunming 650504 Yunnan, China Yunnan University Kunming China; 4 Yunnan Academy of Biodiversity, Southwest Forestry University, Kunming, Yunnan 650224, China Southwest Forestry University Yunnan Malaysia; 5 Center for Interdisciplinary Biodiversity Research & College of Forestry, Shandong Agricultural University, Tai’an, Shandong 271018, China Shandong Agricultural University Shandong China

**Keywords:** *Cephalostachyum*, epitype, genome skimming, *Neomicrocalamus*, new combination, new species, new synonyms

## Abstract

*Melocalamus* is a genus of paleotropical woody bamboos distributed in Southeast Asia, characterised by its complex taxonomic history. Currently, the taxonomic status of several species within *Melocalamus* remains unresolved, primarily due to incompleteness of vegetative specimens for identification, coupled with scarcity of floral and fruit specimens and phylogenetic discordance. To address these issues, we developed a genome skimming dataset with multiple samples per species of *Melocalamus* through the Skmer approach. Genetic and morphological evidence supports the transfer of *M.elevatissimus* to *Cephalostachyum*, with a new combination, *C.elevatissimum*. Based on a comprehensive evaluation that integrates morphological characters, molecular data and geographic distribution, we propose treating *M.utilis* as a synonym of *M.orenudus* and provide the description of its inflorescence and an epitype of it for the first time. Our results further indicate that *M.ningmingensis* is a synonym of *Neomicrocalamusprainii*. We also described a new species, *M.guangxiensis* from Ningming, Guangxi, based on morphological and molecular evidence. Our results demonstrate both the efficiency and reliability of the Skmer approach in species discrimination while emphasising the importance of integrating morphological characters with genomic data for accurate species classification in *Melocalamus*.

## ﻿Introduction

*Melocalamus* Benth. is a genus of the subtribe Bambusinae in continental Southeast Asia, which belongs to the paleotropical woody bamboos clade (PWB) (Poaceae, Bambusoideae) (Zhou et al. 2017; Liu et al. 2024a). The genus was established by Bentham with *Melocalamuscompactiflorus* (Kurz) Benth (based on *Pseudostachyumcompactiflorum* Kurz) as its type (Bentham 1883). *Melocalamus* has long been treated as a monospecific genus (Clayton and Renvoize 1986) because of limited fieldwork and scarcity of herbarium specimens. However, recent studies indicate that it comprises about 14 species (Soreng et al. 2022) with additional new combinations and newly-described species (Liu et al. 2023; Chen et al. 2025). The identification of bamboo species mainly relies on vegetative characters; thus, in the absence of flowering specimens, distinguishing *Melocalamus* from morphologically similar genera such as *Dinochloa* Buse (Liu et al. 2023) and *Maclurochloa* K.M. Wong (Wong 1993) becomes particularly challenging, because these genera are also climbing bamboos in Southeast Asia with one main branch as thick as the main culm. With the advancement of molecular phylogenetics, phylogenomics (Guo et al. 2023) and increased field investigations, it is now feasible to accurately identify new species and taxonomic combinations within *Melocalamus* (Zhou et al. 2017; Qin et al. 2019; Liu et al. 2023), even leading to the recognition of a new genus within the PWB clade (Haevermans et al. 2020).

Species of *Melocalamus* are distributed in humid and warm environments across tropical and subtropical regions of China and mainland Southeast Asia, with documented occurrences in south China (Yunnan, Hainan and Guangxi), Myanmar, Thailand, Laos and Vietnam. According to *Flora Reipublicae Popularis Sincae* (Keng and Wang 1996) and *Iconographia Bambusoidearum Sinicarum* (Yi et al. 2008), the diagnostic morphological characters of *Melocalamus* can be summarised as follows: climbing habit; multiple branches with one dominant branch as thick as main culm; prominent nodes, with a ring of powder/tomenta above and below the sheath scars; pseudospikelets bearing 2–3 florets with a sterile terminal floret that forms head-like inflorescences.

Although classified within the distantly-related PWB subtribe Melocanninae (Zhou et al. 2022), *Cephalostachyum* Munro exhibits vegetative convergence with *Melocalamus* particularly amongst climbing species such as *Cephalostachyumscandens* Bor and *C.mannii* (Gamble) Stapleton & D.Z. Li. The dominant branch of these two species is well-developed and as thick as the main culm, allowing it to substitute for the main culm for climbing. In addition, both genera have overlapping distributions in China. However, *Cephalostachyum* can be distinguished from *Melocalamus* by its reproductive traits such as the prominent bracts below the pseudospikelets, each pseudospikelet containing only one floret and nut-like caryopsis with a persistent style base, supplemented by vegetative differences including glabrous nodes and generally thinner culm walls.

The taxonomic validity of several *Melocalamus* species remains unresolved. *Melocalamuselevatissimus* Hsueh & T.P. Yi, published in 1983 (Yi 1983), is the only species of *Melocalamus* reported in Xizang. It exhibits morphological affinities to *Cephalostachyum*; however, there has been limited collection and rare research since its publication. Furthermore, *Melocalamusgracilis* W.T. Lin (type specimen: Feipeng Chen 4726, deposited at CANT) was described from vegetative material collected in Ningming, Guangxi (Lin 1993). As it is a later homonym of *M.gracilis* R.B. Majumdar (Karthikeyan et al. 1989), which is illegitimate according to the Code (Turland et al. 2025), it was renamed by Ohrnberger (1997) as *M.ningmingensis*. Liu et al. (2023) temporarily treated it as *incertae sedis*. Based on careful examination of the type specimen, we find that it shares significant similarities to *Neomicrocalamusprainii*. Additionally, the vegetative traits of *Melocalamusorenudus* and *M.utilis* are extremely similar. In short, the taxonomic status of these species needs further investigation.

To address above questions, we conducted extensive investigation into the species of *Melocalamus* in China following our previous work (Liu et al. 2023; Chen et al. 2025). We collected multiple individuals per species and detailed morphological comparisons to verify the identities within *Melocalamus* species. A genome skimming dataset was developed using Skmer analysis to further support morphological identifications. The integration of morphological and genetic analyses enabled us to revise the taxonomic position of both *M.elevatissimus* and *M.ningmingensis* and to assess the relationships between *M.orenudus* and *M.utilis*. In addition, we provide a detailed description of inflorescence for *M.orenudus* and describe as well as illustrate a new species of *Melocalamus* from Guangxi, China.

## ﻿Material and methods

### ﻿Morphology observation

To determine the taxonomic status of *Melocalamuselevatissimus*, we reviewed relevant literature before carrying out fieldwork in the type locality and compared specimens in the Herbarium of Kunming Institute of Botany, Chinese Academy of Sciences (KUN). We measured a batch of diagnostic characters including branch complement, culm leaves, internodes, nodes and foliage leaves to compare it with two related species (*M.compactiflorus* and *Cephalostachyumlatifolium*) with living materials, herbarium specimens and images whenever available.

Our previous work found that an individual of *Melocalamusutilis* was clustered with *M.orenudus* (Liu et al. 2023). To check the taxonomic status, we collected additional living materials and specimens of these two species in Hainan and observed relevant specimens from KUN and Herbarium of Sun Yat-sen University (SYS).

We also carried out a field survey of *Melocalamusningmingensis* in the type locality, Ningming, Guangxi in 2023 and carefully examined the type specimen (Feipeng Chen 4726) in the Herbarium of South China Agricultural University (CANT) to determine the status of the taxon *incertae sedis* in Liu et al. (2023).

### ﻿Molecular sampling and analyses

#### ﻿Taxon sampling

A total of 58 individuals were sampled, including 42 individuals of *Melocalamus* (12 species and one variety collected in China, Myanmar and Thailand). To solve the existing taxonomic problems within *Melocalamus*, 11 individuals of nine taxa from subtribe Bambusinae were chosen as closely-related species and five individuals of four species from subtribe Melocanninae as outgroups following Zhou et al. (2017) and Liu et al. (2024a). Amongst them, genome sequencing data of *Dendrocalamuslatiflorus* Munro, *D.sinicus* L.C. Chia & J.L. Sun and *Melocannabaccifera* (Roxb.) Kurz were downloaded from BambooBase (https://bamboo.genobank.org/) (Liu et al. 2024b) and GenBank (https://www.ncbi.nlm.nih.gov/genbank/) (ID: SRR11805848, SRR25459136 and SRR25498978). All examined and voucher specimens are deposited in the KUN Herbarium unless specified. Sample information is detailed in Suppl. material 1: table S1.

#### ﻿DNA sequencing and data cleaning

Silica-gel dried leaves or specimens were used for DNA extraction, library preparation and sequencing. Skmer analysis can achieve satisfactory results using only 1× sequencing depth (Sarmashghi et al. 2019). Given the genome size of *Melocalamus* species is below 2 Gb, we extracted a volume of 2 GB dataset from the sequencing data using Seqkit v.2.3.0 (Shen et al. 2016), with single-end sequencing data comprising 1 GB (approximately 8,000,000 reads). Fastp 0.21.0 (Chen et al. 2018) was used for quality control of raw reads with the default parameters. The sequencing quality of samples are shown in Suppl. material 1: table S2.

### ﻿Dataset construction and phylogenetic analyses

We performed independent Skmer (Sarmashghi et al. 2019) analyses by utilising two distinct datasets: one included all clean reads, while the other excluded plastid and mitochondrial reads aiming to evaluate whether the inclusion of organellar genomes would influence the final topological structure. Before initiating analysis, Bowtie2 v.2.3.4.1 (Langdon 2015) was utilised to establish plastid and mitochondrial reference for the purpose of removing plastid and mitochondrial sequences from the sequencing data, plastome reference from *Melocalamusarrectus* T.P. Yi (MK679766.1) and mitochondrial genome sequences of *Ferrocalamusrimosivaginus* T.H. Wen (Ma et al. 2012). Then BBMerge (Bushnell et al. 2017) was used to merge overlapping read pairs with default parameter settings. We used “skmer reference” and “skmer subsample” commands to obtain a main estimate distance matrix and 100 sub-replicates. By utilising the “skmer correct” command, we performed correction of 100 subsampled distance matrices ultimately obtaining correct genomic estimates. Then, these standard square distance matrices were converted to PHYLIP format. FastME 2.1.6.4 (Lefort et al. 2015) was used to infer phylogenies from the reformatted distance matrices. We used RAxML v.8.2.12 (Stamatakis 2014) to construct an extended majority rule consensus tree with the model “GTRCAT”. The PHYLIP format subsampled distance matrices for the analysis reported in this paper were deposited in the Science Data Bank at https://doi.org/10.57760/sciencedb.26005.

## ﻿Results

### ﻿Morphological comparison

**Figure 1.**
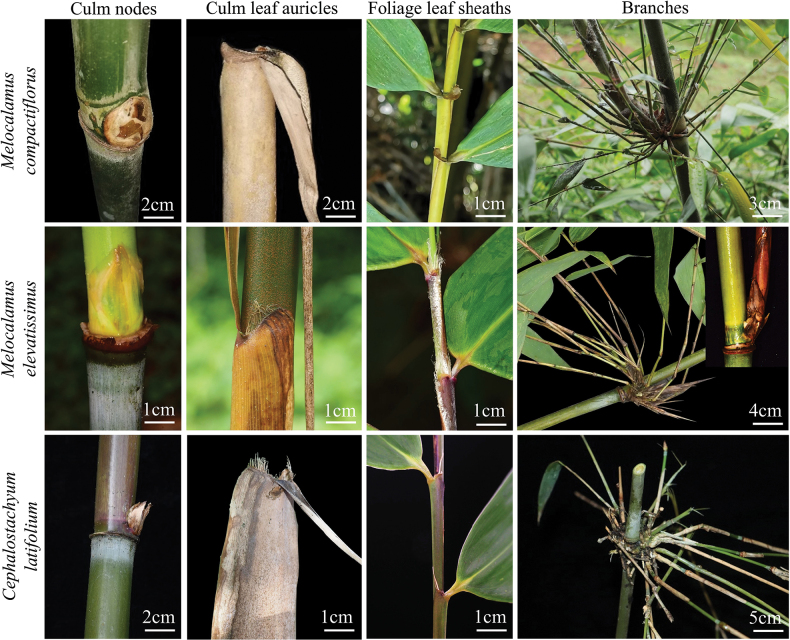
Comparison of morphological characters amongst *Cephalostachyumlatifolium*, *Melocalamuselevatissimus* and *Melocalamuscompactiflorus*.

Morphologically, *Melocalamuselevatissimus* is similar to species of *Melocalamus* in terms of vegetative characters, such as climbing habits, branches several to many, one dominant branch as thick as main culm, with white powder under nodes and it somewhat resembles *M.compactiflorus*. However, *M.elevatissimus* can be distinguished from *M.compactiflorus* by the following suite of diagnostic characters: (1) culm leaves lack auricles, with apices protruding into thin projections (1–2 cm on each side); (2) culm leaf blades are lanceolate, characterised by a base that does not contract into a rounded shape; (3) foliage leaf sheaths possess elongated oral setae extending to the mid-region, without auricles. However, *M.elevatissimus* is more similar to *Cephalostachyum* in glabrous internodes (without siliceous), smooth nodes, without white powder/tomenta above the nodes and culm leaf sheaths with obvious transverse veins. After comparing it with all the species of *Cephalostachyum*, we found that *M.elevatissimus* is mostly similar to *Cephalostachyumlatifolium*, mainly reflected in thin culm leaf sheaths with obvious longitudinal ribs abaxially and with obvious oral setae. However, *M.elevatissimus* usually has one main branch as thick as culm; foliage leaf sheaths with fimbriate grey-white long oral setae, margins with fringed cilia; while *C.latifolium* usually has many subequal branches, sometimes with 1–2 thicker ones, without main branches; foliage leaf sheaths with caducous white straight oral setae, margins without cilia. We presented/summarised the morphological character differences of *M.compactiflorus*, *M.elevatissimus* and *C.latifolium* in Fig. 1 and Table 1, respectively.

**Table 1.** Morphological comparison amongst *Melocalamuscompactiflorus*, *Melocalamuselevatissimus* and *Cephalostachyumlatifolium*.

Characters/Species		*Melocalamuscompactiflorus*	*Melocalamuselevatissimus*	*Cephalostachyumlatifolium*
Culm leaves	Sheaths	Persistent; thickly leathery; with white powder abaxially; without obvious longitudinal veins and transverse veins	Persistent; leathery; with light-yellow spiny hairs abaxially; with obvious longitudinal veins and transverse veins	Deciduous; leathery, with sides papery; densely brown hairs abaxially; with obvious longitudinal veins and transverse veins
	Apex	Truncate, flat	With thin projections 1–2 cm on each side	Round, projecting upward on both sides
	Ligules	Entire, narrow	Depressed, ca. 0.1 cm	Narrow
	Auricles	Crescent-shaped, reflexed	Absent	Inconspicuous
	Oral setae	Absent	Caducous grey-white long hairs	Caducous fimbriate long hairs
	Blades	Circular at the base, reflexed	Lanceolate, reflexed or erect	Lanceolate, reflexed or erect
Foliage leaves	Apex	Flat	Protruding	Protruding, circular
	Ligules	Entire, narrow	Depressed, ca. 0.1 cm	Truncate, short
	Auricles	Crescent-shaped, reflexed	Absent	Inconspicuous
	Oral setae	Absent	With curly fimbriate grey-white long oral setae	Caducous, with white straight oral setae
	Blades (length × width)	15–25 cm × 3–3.5 cm, lanceolate	17–28 cm × 3–9 cm, oblong-lanceolate	25–30 cm × 8–10 cm, ovate-elliptic
Culms	Walls	1–2.5 cm in diam.; wall thick to solid	1.5–3 cm in diam.; wall 0.3–0.4 cm	3.5 cm in diam.; wall 0.2–0.3 cm
	Internodes	Rough, with siliceous	Glabrous	Glabrous
	Nodes	Prominent, with tomenta	Smooth, glabrous	Smooth, glabrous
Branches		Many, with one dominant branch	Many, with one dominant branch	Many, without dominant branch

We summarised the distinctions outlined in the literature regarding *Melocalamusutilis* and *M.orenudus*. Both the morphological characteristics described in original literature (McClure 1940) and our previous work (Liu et al. 2023) concur that the primary morphological differences between the two species are summarised as follows: 1) *M.utilis* has narrow culm leaf auricles, while culm leaf auricles are absent in *M.orenudus*; 2) The culm leaf sheaths of *M.utilis* have white spiny hairs, while the culm leaf sheaths of *M.orenudus* have appressed white pubescent; 3) The foliage leaf sheaths of *M.utilis* are slightly striped and glabrous, while the foliage leaf sheaths of *M.orenudus* have pubescent and white powder. Through investigation in Lingshui and Baoting, Hainan, the type localities and re-examination of specimens, we found that the two species exhibit high similarity: 1) The culm leaf auricles of *M.utilis* are also frequently absent; 2) Overall, the culm leaf sheaths of both species are powdery and covered with white pubescence; 3) The foliage leaf sheaths of both species are slightly striped and glabrous in specimens and the tender foliage leaf sheaths are covered with easily deciduous short hairs and white powder in living materials.

As for *Melocalamusningmingensis*, due to the type specimens providing limited information, especially the incomplete culm leaf sheaths, we could neither find the blade information of culm leaf sheaths in the protologue of Lin (1993) nor from the type specimen (Feipeng Chen 4726). In addition, we could not observe the branch sheaths described in the part 6 of fig. 1 from the type specimen either (Lin 1993). It is difficult to undertake further comparison in morphology. Through detailed examination of the original descriptions and the meticulous observation of the type specimen, we have determined that only the common climbing habit and the branches exhibit similarities to *Melocalamus* and neither culm diameter (only 5–8 mm) nor glabrous nodes/culms match the characteristics of *Melocalamus*. Instead, these features are more consistent with those of *Neomicrocalamus*. In addition, according to the type specimens, there is no ring of powder and/or tomenta above and below the sheath scars, internodes smooth, culm leaf sheaths with brown spotted abaxially. These characters are matched well with *Neomicrocalamusprainii* (Gamble) Keng f. (Keng 1983), rather than *N.andropogonifolius* (Griff.) Stapleton (Stapleton 1994) or *N.dongvanensis* T.Q. Nguyen (Nguyen 1991). Vegetatively, *N.andropogonifolius* has hollow culms and glabrous culm leaf sheaths without bristle, while Feipeng Chen 4726 has solid culms and culm leaf sheaths with brown spots and small bristles. *N.dongvanensis* has upright culms, while Feipeng Chen 4726 has drooping culms.

**Figure 2.**
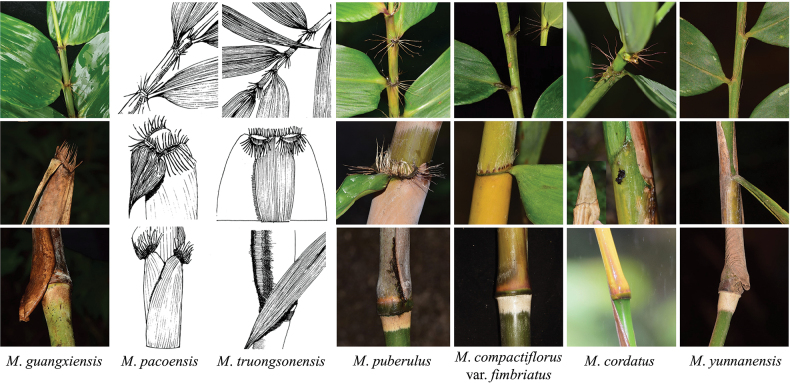
Diagnostic characters of foliage leaves and culm leaves amongst *M.guangxiensis* and closely-related species (The bases of culm sheaths are missing in *M.pacoensis* and *M.truongsonensis* because there are no relevant pictures available (Nguyen and Tran 2010)).

During our field survey in the type locality of *Melocalamusningmingensis* within the Longrui Nature Reserve, Ningming, Guangxi, we collected a potentially new species named *Melocalamusguangxiensis* D.Z. Li & J.X. Liu (collection number: Xuzc2023109, PX001) which possess key diagnostic features of *Melocalamus*, including the prominent nodes, with a ring of tomenta above and below the sheath scars, many branches with one dominant branch replacing the main culm. The characters of foliage leaf auricles with radiate oral setae which are similar to *Melocalamuspuberulus* (McClure) D.Z. Li & J.X. Liu (Liu et al. 2023), *M.cordatus* (T.H. Nguyen Wen & Q.H. Dai) D.Z. Li & M.Y. Zhou (Zhou et al. 2017), *M.pacoensis* H.N. Nguyen & V.T. Tran (Nguyen and Tran 2010), *M.truongsonensis* H.N. Nguyen & V.T. Tran (Nguyen and Tran 2010) and M.compactiflorusvar.fimbriatus (Hsueh & C.M. Hui) D.Z. Li & Z.H. Guo. In addition, the characters of culm leaf ligules with fringed long setae at the apex are mostly similar to *M.pacoensis*, *M.truongsonensis*, *M.puberulus* and M.compactiflorusvar.fimbriatus, but could be easily identified with the different culm leaf blades: only the culm leaf blades of *M.guangxiensis* are not constricted to round shape or narrow at the base part. The unique character of *M.guangxiensis* is the base of the outer margin of culm leaf sheaths with a membranous projection, ca. 1–2 cm. Apart from the presence of an inconspicuous membranous projection at the base of the outer culm sheath margin in *M.yunnanensis*, this feature has not been observed in any other *Melocalamus* species. The comparation of culm leaves and foliage leaves within *M.guangxiensis* and the above six taxa is presented in Fig. 2 and Table 2.

**Table 2.** A comparison of culm leaves and foliage leaves amongst *Melocalamusguangxiensis* and closely-related species.

Characters /Species		*M.guangxiensis*	*M.pacoensis*	*M.truongsonensis*	*M.puberulus*	M.compactiflorusvar.fimbriatus	*M.cordatus*	*M.yunnanensis*
Culms (cm)	Culms diameter	1.0–2.0	1.8–2.3	2.0–3.0	2.0–3.5	2.0–4.0	2	(1–)2.0–3.0
	Culm walls thickness	0.4–0.5	0.8	solid	0.4	0.8–1.3	0.5	0.5–0.7
	Internodes length	50–80	80–85	58–60	30–50	25–35(–45)	40–60	40–60
Culm leaf sheaths (cm)	Base	Base of outer margin with a membranous projection, ca. 1–2 cm	Base without a projection	Base without a projection	Base without a projection	Base without a projection	Base without a projection	Base of outer margin with an inconspicuous projection
	Outer surface	With white powder and brown hairs	With smooth, fugacious, black hairs	With dense, soft, appressed, black hairs	With white powder and black hairs	With caducous white hairs	With yellow pubescent	With brown cilia
	Auricles	Erect or recurved, wavy, narrow, with several fimbriate long oral setae	4–4.5 × 0.5–0.6 cm, rounded, serrate, with 2 cm long stiff hairs	0.5–0.8 × 0.6–0.7 cm, stiff, thick, deflexed, with two lines of 2.2 cm long hairs.	Conspicuous, reflexed, wrinkled, with radiated oral setae, 1.5–2.5 cm long	Inconspicuous	Ovate, with fimbriate oral setae or inconspicuous	Absent
	Ligules	0.2–0.3 cm, uniformly serrated, apex with fimbriate hairs, 1–1.5 cm long	Rounded, with sparse, 1.2 cm long hairs	0.2 cm, with fugacious hairs, become serrate after shedding	0.15–0.4 cm, apex wide arched, fimbriate	0.2–0.5 cm, prominent, with 0.8–1 cm fimbriate hairs	0.1 cm, edge with cilia	0.1 –0.3 cm, apex truncate or prominent
	Blades	6–8 × 0.7–0.9 cm, lanceolate	13–19 × 4.5–6.5 cm, triangular, round at the base	4–7 × 0.5–0.8 cm, round/cordate at the base	2–2.5 × 16–18 cm, lanceolate, round/cordate at the base	2–18 × 11.8–2.5 cm, ovate-lanceolate, round/cordate at the base	25–32 × 9–10 cm, ovate-lanceolate, with equal width of sheath apex	(4–)7–10.5 × 0.8–1.0 (–1.3) cm, ovate-lanceolate
Foliage leaf sheaths (cm)	Auricles	Sickle-shaped, with radiated oral setae	0.2–0.8 cm, undulated, with sparse, stiff oral setae 2.5 cm long	0.1–0.3 cm, curved outwards, with dense, stiff, oral setae 1.5 cm long	Conspicuous, oral setae radiated, 1–1.5 cm long	Absent	Sickle-shaped, oral setae, 1.3 cm long	Absent
	Ligules	0.1–0.2 cm, apex truncate or with inconspicuous serrated, with fimbriate long hairs, 1 cm long	0.2 cm, with sparse stiff hairs	Short	0.1–0.2 cm, fimbriate, apex truncate	0.1 cm, apex serrated	0.1 cm	Inconspicuous
Distribution		Guangxi, China	Vietnam	Vietnam	Yunnan, China	Yunnan, China	Hainan, China	Yunnan, China

Over-reliance on some diagnostic characters while overlooking other morphological characters could result in misclassifications. In the case of ambiguous *Melocalamus* species, the identification of *M.elevatissimus* and *M.ningmingensis* were mainly based on the presence of a branch as thick as the culm and their climbing habit, which led to the incorrect identification as *Melocalamus*. However, our molecular and morphological comparison results all support the species that should belong to *Cephalostachyum* and *Neomicrocalamus*, respectively, because these three genera all have main branches and are all climbing bamboo species. However, the most conspicuous distinctions amongst these three genera are based on the character on culms and culm leaves. We have summarised these key diagnostic characters in Table 3.

**Table 3.** A comparison of key morphological characters amongst *Cephalostachyum, Melocalamus* and *Neomicrocalamus*.

Characters/Genus		*Cephalostachyum*	*Melocalamus*	*Neomicrocalamus*
Culm	Walls	Hollow	Solid or sub-solid or hollow	Solid or sub-solid
	Internodes	Glabrous	Rough, with siliceous	Glabrous
	Nodes	Smooth, glabrous	Prominent, with tomenta	Smooth, glabrous
Culm leaves	Auricles and oral setae	Prominent	Prominent or with highly elevated shoulders or absent	Absent
	Blades	Lanceolate	Lanceolate, round/cordate at the base	Conical

### ﻿Skmer tree of *Melocalamus* species with uncertain taxonomic status

The total length of sequences ranges from 1,126,690 to 2,315,977 kb. After removing plastid and mitochondrial reads, the sequence length ranges from 1,095,340 to 2,254,224 kb.

**Figure 3.**
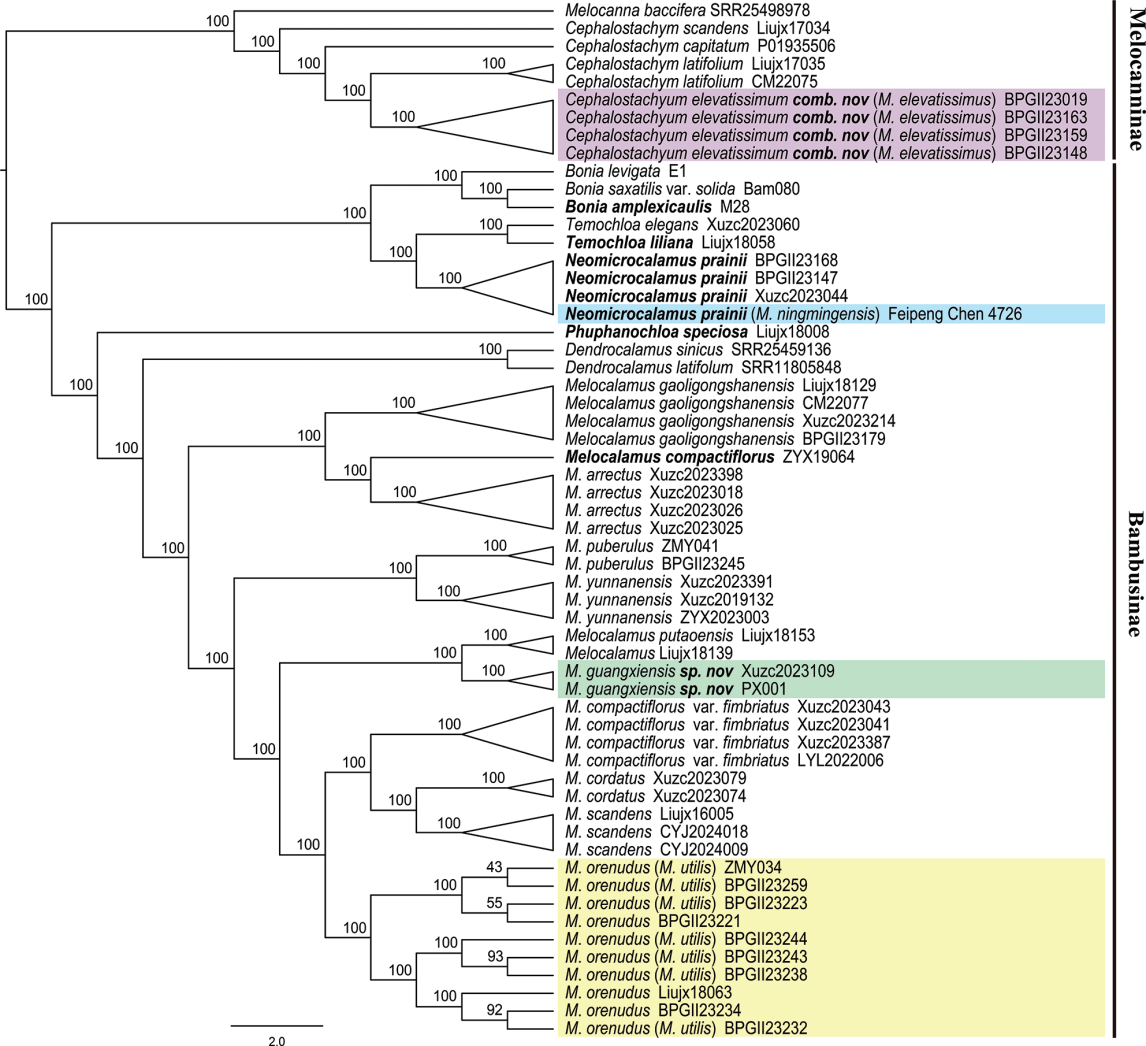
The topology of the sampled *Melocalamus* with its related taxa, based on the Skmer analysis with all reads. Coloured boxes indicate the doubtful species and the species names in parentheses indicate the corresponding names before being revised in this study. Types of generic names are in bold.

The topological structures constructed from the two datasets are completely identical, which divide all individuals into two main clades: Melocanninae and Bambusinae (Fig. 3 and Suppl. material 2). Four individuals of *Melocalamuselevatissimus* form a monophyletic clade, which is closely related to *Cephalostachyumlatifolium*, indicating that this species is a member of *Cephalostachyum* (purple marked in Fig. 3), based on the Skmer tree. The type specimen of *Melocalamusningmingensis* (Feipeng Chen 4726) (blue marked in Fig. 3) is clustered with *Neomicrocalamusprainii*, which is consistent with its morphological characteristics. The other 37 individuals of *Melocalamus* form a well-supported monophyletic clade. Putative conspecific individuals are grouped in their respective clades, except for the seven individuals of *M.utilis* and three individuals of *M.orenudus* (yellow marked in Fig. 3), which together comprise a highly-supported subclade. The interspecific boundaries are not clear for the two species, rendering our proposal to combine *M.utilis* into *M.orenudus.* The new species *M.guangxiensis* is closely related to *M.putaoensis* D.Z. Li & J.X. Liu (Chen et al. 2025) but can be easily distinguished by the differences in morphological characteristics: the culm sheaths of *M.guangxiensis* possess a membranous projection, while those of *M.putaoensis* lack this structure; *M.guangxiensis* exhibits wavy auricles with several long fimbriate oral setae, whereas *M.putaoensis* lacks both auricles and oral setae.

## ﻿Discussion

### ﻿Skmer approach is efficient and reliable in species identification of *Melocalamus*

Based on the distance matrices, we reconstructed a stable Skmer tree of *Melocalamus* with high support values. The monophyly of *Melocalamus* and all the 10 revised taxa with multiple samples (except *M.compactiflorus* with only one individual) has been confirmed, consistent with previous results obtained from nuclear genes, including SNPs derived from dd-RAD data (Liu et al. 2020; 2023; 2024a) and syntenic nuclear genes (Chen et al. 2025). However, the phylogenetic tree constructed from plastid loci did not to support the monophyly of *Melocalamus* and exhibited low stability in topological structure (Zhou et al. 2017; Liu et al. 2020).

Zhang et al. (2023) compared species identification rates amongst different barcodes, based on a dataset of 50 species of *Cymbidium* Sw. (Orchidaceae). Through Maximum Likelihood (ML) tree analysis, the identification rate, based on the plastome, increased to 68%, compared to 58% for the standard barcodes (*rbcL* + *matK* + *trnH*-*psbA*) and the Skmer approach enhanced the identification rate to 72%. In another study focusing on 13 taxa of *Schima* Reinw. ex Blume (Theaceae) (Duan et al. 2024), it was found that the standard barcoding markers (*rbcL* + *matK* + *trnH*-*psbA*) lacked sufficient resolution, while the plastome raised the identification rate to 27.27% and the Skmer approach achieved an identification rate of 60%. Notably, the topological structure constructed using the Skmer approach correlated well with both morphological characteristics and geographical distribution within *Schima*. The two studies suggest that, based on a large amount of nuclear data, the Skmer approach enhances the accuracy and sensitivity in species identification when compared to the standard barcoding markers and plastome data.

In addition, the Skmer method eliminates the plastome assembly and sequence alignment steps by enabling intergenomic distances calculations using low-coverage sequencing data while demanding minimal computational resources. The confirmation of monophyly in *Melocalamus* species along with improved resolution of interspecific relationships in this study underscores that the Skmer approach is an efficient and reliable strategy for species identification within *Melocalamus*.

### ﻿Revision of species with uncertain status in *Melocalamus*

Based on the stable topology of *Melocalamus*, reconstructed using the Skmer approach, *Melocalamuselevatissimus* and *Cephalostachyumlatifolium* form a sister group in a separate clade, while all other samples form another clade. However, *Melocalamusningmingensis* and three individuals of *Neomicrocalamusprainii* exhibit the closest relationship, rather than with other species of *Melocalamus*, which correspond well with our morphological comparison. Within the *Melocalamus* clade, individuals of *M.orenudus* and *M.utilis* mix with one another, but together cluster into a highly-supported subclade. The potentially new species, *Melocalamusguangxiensis* is not related to the type specimen of *M.ningmingensis* and can be distinguished from other species of *Melocalamus*, confirming its identity suggested by morphological analysis. Therefore, we treat these doubtful species as follows.

### ﻿Taxonomic treatment

#### ﻿ Cephalostachyum elevatissimum

Taxon classificationPlantaePoalesPoaceae

(Hsueh & T.P. Yi) D.Z. Li, Y.X. Zhang & Y.J. Chen comb. nov.

E5F2056E-8786-53C2-B0FF-827FC2E0471B

urn:lsid:ipni.org:names:77364889-1

**Figure 4.**
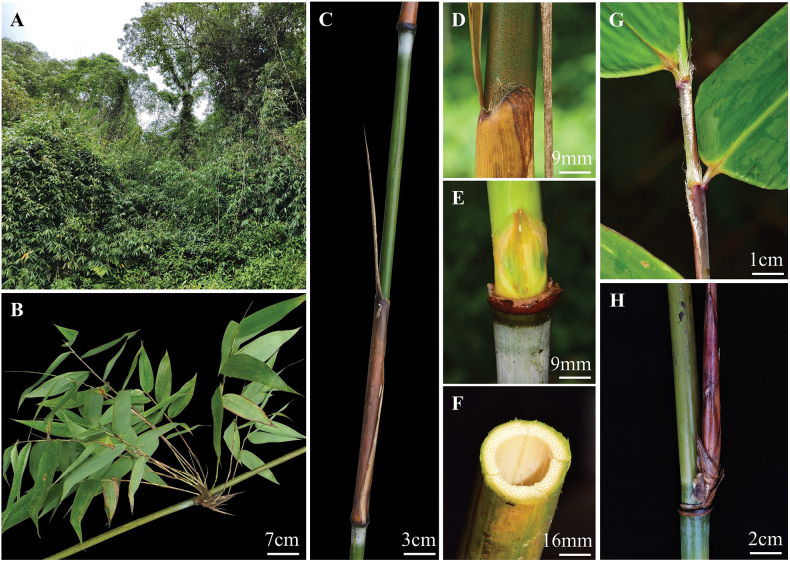
*Cephalostachyumelevatissimum* (Hsueh & T.P. Yi) D.Z. Li, Y.X. Zhang & Y.J. Chen. **A.** Habitat; **B, H.** Branch complement; **C.** A complete internode with culm leaf sheath; **D.** Culm leaf sheath apex; **E.** Node and culm bud; **F.** Transection of main culm; **G.** Foliage leaf sheath.

Fig. 4 Local names. Sang Rong, Ping Er. Chinese name. “西藏空竹” (xī zàng kōng zhú).

≡ Melocalamuselevatissimus Hsueh & T.P. Yi, J. Bamboo Res. 2(1): 28, 1983.

##### Type.

China • Xizang: Linzhi City, Motuo County, Beibeng Town, Deergong, alt. 940–2000 m, 15 Aug 1977, *T.P. Yi 77183* (holotype, SIFS!, without barcode).

##### Description.

Perennial. Rhizomes pachymorph, short-necked. Culms apically scrambling, ca. 17 m long, 1.4–3 cm in diam.; internodes terete, glabrous, 40–90 (–120) cm long, wall 0.2–0.4 cm thick; nodes flat, glabrous; white powdery below the nodes; sheath scars prominent. Primary branch buds solitary, ovate-elliptical, compressed. Branches many, subequal or occasionally with a dominant branch replacing main culm. Culm leaves tardily deciduous, 24–38 cm × 8–12 cm, 2/5 to 1/2 as long as the internodes; sheaths leathery, long-triangle, with appressed light-yellow spiny hairs abaxially, margins glabrous, apex U-shaped and projecting upward sides, papery, with fimbriate grey-white long oral setae; auricles absent; ligules truncate, short, ca. 1 mm in length, margin extremely shortly ciliolate or subglabrous; blades linear-lanceolate, erect or reflexed, 5–30 cm × 1–1.9 cm. Foliage leaves 6–9 per ultimate branch; sheaths 8–16 cm long, abaxially glabrous, apex with fimbriate grey-white long oral setae, 4–11 mm long; margins with fringed cilia, 6–15 mm long; auricles absent; inner ligules truncate, dark-purple, ca. 1 mm in length; outer ligules present, truncated; blades oblong-lanceolate, 17–28 cm × 3–9 cm, with white pubescence abaxially, margins coarse, secondary veins 5–12 paired. Inflorescence and caryopsis unknown.

##### Phenology.

New shoots May to August.

##### Additional specimens examined.

China • Xizang: Linzhi City, Motuo County, alt. 1995 m, 15 Jul 2021, *Z.Y. Xiahou XHZY2021003*; • ibid., 30°1'7.097"N, 94°59'44.77"E, alt. 2061 m, 13 Jun 2023, *P.F. Ma et al. BPG II 23141*; • ibid., 29°38'48.75"N, 95°29'4.70"E, alt. 1903 m, 14 Jun 2023, *P.F. Ma et al. BPG II 23148*; • ibid., 29°14'37.071"N, 95°11'16.95"E, alt. 1302 m, 15 Jun 2023, *P.F. Ma et al. BPG II 23159*; • ibid., 29°10'50.10"N, 95°8'36.56"E, alt. 1736 m, 15 Jun 2023, *P.F. Ma et al. BPG II 23163*; • Xigaze City, Yadong County, Xiayadong, alt. 1625 m, 16 Jul 2021, *J.D. Ya et al. 21CS20510*; • ibid., 27°14'4.89"N, 89°1'2.89"E, alt. 1684 m, 6 Jun 2023, *P.F. Ma et al.*, *BPG II 23019*.

##### Note.

**Figure 5.**
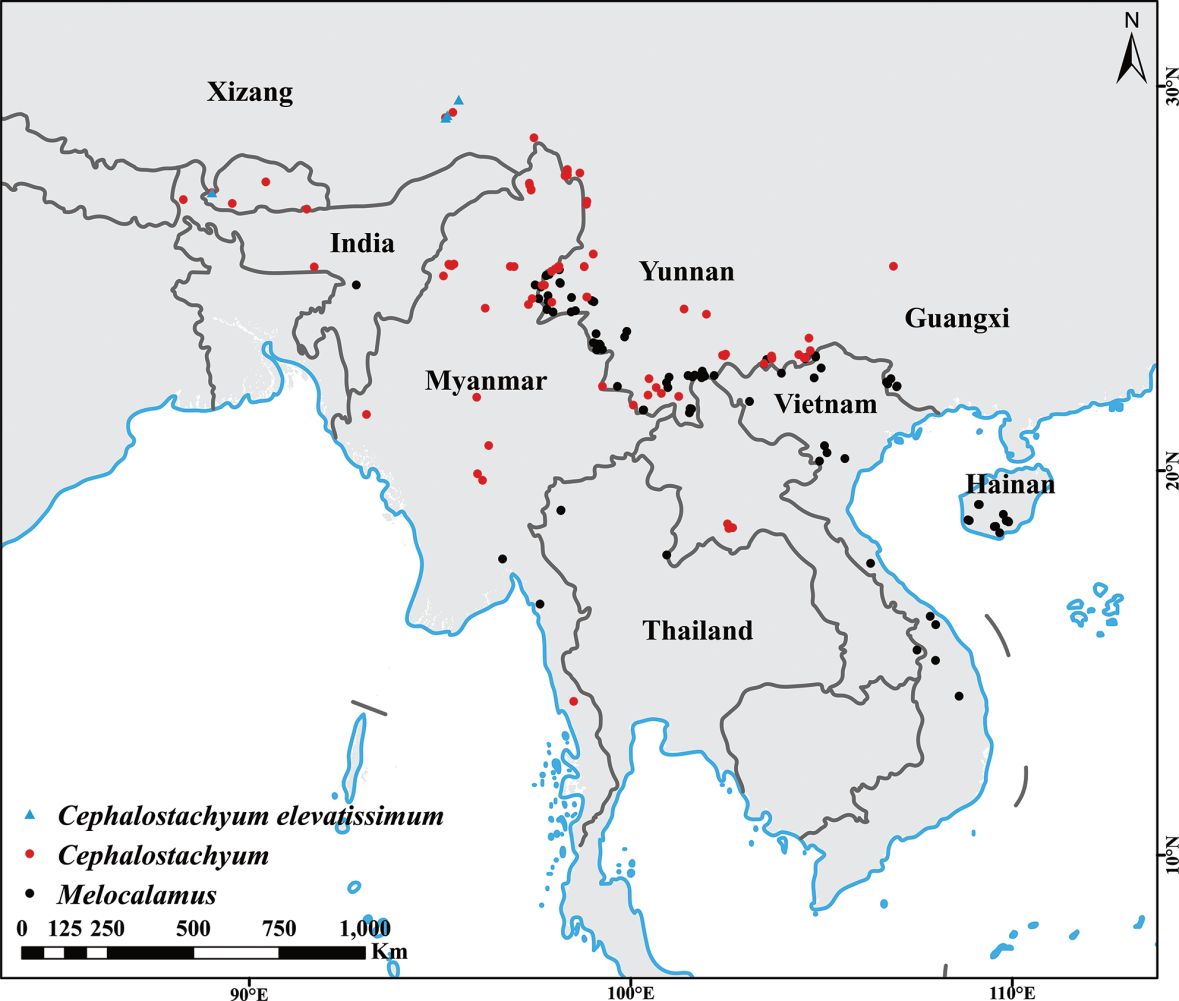
Geographical distribution variations of *Cephalostachyumelevatissimum* (blue dots), other species of *Cephalostachyum* (red dots) and species of *Melocalamus* (black dots).

*Melocalamuselevatissimus* was the first species of *Melocalamus* published in China (Yi 1983), which has a narrow distribution area in southern Xizang, confined to Motuo and Yadong, as well as the Yarlung Zangbo Grand Canyon National Nature Reserve, Xizang (Wu et al. 2022). *Cephalostachyumelevatissimum* does not overlap with the distribution area of other *Melocalamus* species (distributed in the Yunnan, Guangxi and Hainan Provinces of China, as well as Thailand, Myanmar, Vietnam and other Southeast Asian countries), but shares a common range with *Cephalostachyum* (Fig. 5). In addition, *Melocalamus* are predominantly found at altitudes ranging from 200 to 1300 m, while *C.elevatissimum* in Xizang mainly inhabits areas in higher altitudes (mainly 1300 to 2060 m).

#### ﻿ Melocalamus orenudus

Taxon classificationPlantaePoalesPoaceae

(McClure) D.Z. Li & J.X. Liu.

8FE7C3C5-4121-57B3-8BE4-BBE766C3E81B

**Figure 6.**
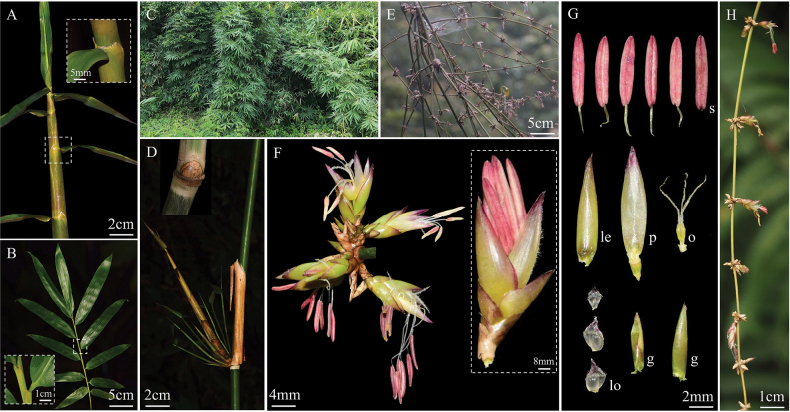
*Melocalamusorenudus* (McClure) D.Z. Li & J.X. Liu. **A.** New shoot, showing sheath auricle and ligule; **B.** Foliage leaves, showing foliage leaf sheaths; **C.** Habitat; **D.** Culms with culm leaf sheaths, branch complement and node with bud; **E, F, H.** Inflorescence and pseudospikelets; **G.** Stamens (s), lemma (le), palea (p), ovary (o), three lodicules (lo) and two glumes (g).

Fig. 6 Chinese name. “无耳梨籐竹” (wú ěr lí téng zhú).

Melocalamus orenudus (McClure) D.Z. Li & J.X. Liu, Plant Divers. 45: 137, 2023.
≡ Dinochloaorenuda McClure, Lingnan Univ. Sci. Bull. 9: 18, 1940.
= Melocalamusutilis (McClure) D.Z. Li & J.X. Liu, Plant Divers. 45: 138, 2023, syn. nov. ≡ Dinochloautilis McClure Lingnan Univ. Sci. Bull. 9: 20, 1940.

##### Type.

China • Hainan: Lingshui County, Chim Shan, Fan Maan Ts’uen and vicinity, 3–20 May 1932, *H. Fung 20230* (holotype, US!, Catalogue No.: 2802829, Barcode: 00065462); • Ledong County, Jianfengling National Nature Reserve, 18°42'21.03"N, 108°52'6.04"E, alt. 826 m, 02 Nov 2023, *M.Y. Zhou, J.X. Liu & Z.C. Xu BPG II 23238* (epitype designated here, KUN!).

China • Hainan, Ling Shui District, Chim Shan, Fan Maan Ts’uen and vicinity, 4–20 May 1932, *McClure 20136* (holotype, US!, Catalogue No.: 2802831, Barcode: 00065464; isotype, US!, Catalogue No.: 2767635, Barcode: 00036336; No.: 2767636, Barcode: 00036337).

##### Description.

Perennial. Rhizomes pachymorph, short-necked. Culms slender, 10–20 m long, 2–5 cm in diam., wall ca. 0.5 cm thick; internodes initially with white pubescence, 30–60 cm long; nodes prominent; sheath scars with a ring of white-yellow tomenta above and below. Branches several to many, with one dominant branch sometimes replacing main culm. Culm leaves shorter than internode; sheaths leathery, with white spiny hairs and powder abaxially, apex slightly concave; auricles narrow, extending upwards or absent; oral setae absent; ligules 1–2 mm in length, denticulate; blades lanceolate, round/cordate at the base, recurved. Foliage leaves 7–15 per ultimate branch; sheaths glabrous, tender sheaths are covered with easily deciduous short pubescence and white powder; auricles narrow or absent; ligules entire, ca. 1 mm in length; blades length 13–21 cm × width 3–4.5 cm, with white pubescence abaxially; margins coarse.

Flowering branches lateral or terminal; internodes 1–15 cm long, with white pubescence. Pseudospikelets lanceolate, top and edge purple, bottom yellow-green, 1–1.2 cm long, several to many clustered on nodes, glomerate, mixed with some small sterile and hay bracts; 3 florets in each psedospikelet with the top one sterile, fertile ones sessile. Glumes 2–3, ovate, 2–5 mm long, 2–3 mm wide, margins smooth. Fertile lemma lanceolate, thick membranous, glabrous, yellow, with top and edge purple, ca. 6–12 mm long. Palea nearly equal as lemma in size, lanceolate, membranous, navicular, 2 keels, margins with white cilia, yellow, with top and edge purple. Lodicules 3, 2 equal-sized, ca. length 2.7 mm × width 1.3 mm, one smaller, ca. length 2 mm × width 1 mm, bottom transparent with top light-purple, margins with long cilia. Stamen 6, distinct, anthers purple-red, sometimes yellow, 5–6 mm long, filament white. Ovary ovate-lanceolate, yellow-green; style 1, stigmas 3, plumose, white. Caryopsis unknown.

##### Phenology.

New shoots May to July.

##### Additional specimen examined.

China • Hainan: Baoting County, Ganzhaling Nature Reserve, 22 Aug 2018, *J.X. Liu & Z.C. Xu Liujx18063*; • Baoting County, Nangai 2 Road, 18°32'47.28"N, 109°31'34.43"E, alt. 354 m, 01 Nov 2023, *M.Y. Zhou, J.X. Liu & Z.C. Xu BPG II 23232*; • ibid., 18°33'5.01"N, 109°33'48.76"E, alt. 238 m, 01 Nov 2023, *M.Y. Zhou, J.X. Liu & Z.C. Xu BPG II 23234*; • Changjiang County, Bawangling National Nature Reserve, 19°7'13.04"N, 109°6'37.25"E, alt. 388 m, 03 Nov 2023, *M.Y. Zhou, J.X. Liu & Z.C. Xu BPG II 23244*; • Lai area, Hung Mo Shan and vicinity, 16 May 1929, *Tsang, Tang & Fung, 17725* (SYS!); • Lingshui County, Chim Shan, Fan Maan Ts’uen and vicinity, 4–20 May 1932, *F.A. McClure 20087* (SYS!); • Lingshui County, Diaoluo Mountain National Nature Reserve, 18°41'21.12"N, 109°52'43.68"E, alt. 612 m, 10 Jul 2017, *M.Y. Zhou et al. zmy034*; • ibid., 24 Aug 2018, *J.X. Liu & Z.C. Xu Liujx18076*; • ibid., 18°42'2.48"N, 109°50'10.38"E, alt. 511 m, 31 Oct 2023, *M.Y. Zhou, J.X. Liu & Z.C. Xu BPG II 23221*; • ibid., 18°40'8.33"N, 109°54'10.75"E, alt. 236 m, 31 Oct 2023, *M.Y. Zhou, J.X. Liu & Z.C. Xu BPG II 23223*; • Ledong County, Jianfengling National Nature Reserve, 18°43'15.09"N, 108°49'54.31"E, alt. 535 m, 02 Nov 2023, M.Y. *Zhou, J.X. Liu & Z.C. Xu BPG II 23243*; • ibid., 18°42'21.02"N, 108°52'6.03"E, alt. 826 m, 02 Nov 2023, *M.Y. Zhou, J.X. Liu & Z.C. Xu BPG II 23238*; ibid., 118°42'0.42"N, 08°51'52.07"E, alt. 753 m, 19 Jun 2024, *M.Y. Zhou, J.X. Liu & Y.J. Chen CYJ2024029* (with fl.); • ibid., 18°40'57.61"N, 108°52'46.39"E, alt. 640 m, 19 Jun 2024, *M.Y. Zhou, J.X. Liu & Y.J. Chen CYJ2024032* (with fl.); • ibid., 18°43'22.15"N, 108°50'3.24"E, alt. 633 m, 21 Aug 2024, *M.Y. Zhou, J.X. Liu & Y.J. Chen CYJ2024052*; • Qiongzhong County, Shipo Village, 18°51'59.97"N, 109°45'52.25"E, alt. 316 m, 4 Nov 2023, *M.Y. Zhou, J.X. Liu & Z.C. Xu BPG II 23259*; • Sanya City, Tianya District, Ming Shan, 26 Aug 2018, *J.X. Liu & Z.C. Xu Liujx18079*, *Liujx18081*; • Sanya City, Tianya District, Ya Lin, 27 Aug 2018, *J.X. Liu & Z.C. Xu Liujx18084*, *Liujx18088*.

##### Note.

*Melocalamusutilis* and *M.orenudus* were initially described as independent species of *Dinochloa* by McClure (1940). Based on molecular phylogeny, Liu et al. (2023) transferred them into *Melocalamus*. Phylogenetically, one sampled individual of *M.utilis* mixed into one clade which made *M.orenudus* paraphyletic in dd-RAD tree (Liu et al. 2023). We sampled more individuals in the Skmer analysis and resulted in a similar topology. *Melocalamusutilis* and *M.orenudus* exhibit high morphological similarity. Furthermore, these two species occupy overlapping distribution ranges, with type specimens collected from the similar location as “Lingshui Dist., Chim Shan, Fan Maan Ts’uen”, in Hainan Province (the type locality now belongs to Sandao Zhen of Baoting County, Fanna Village to Shougong Jianlin). Based on morphological and genetic evidence, as well as geographical distribution data, we conclude that *M.utilis* and *M.orenudus* are conspecific. Since both names were simultaneously published by the same author in the original work and no explicit choice of priority was made (McClure 1940), we propose to treat *M.utilis* as a synonym of *M.orenudus* following the International Code of Nomenclature (ICN) (Turland et al. 2025).

In October 2023, we collected a few flowering individuals of *M.orenudus* in Jianfengling National Nature Reserve, Hainan. We conducted follow-up investigations in this area in June and August 2024, respectively. In total, we found two clumps of *M.orenudus* in the flowering stage that were relatively close to each other and collected three flower-bearing specimens (*BPG II 23238*, *CYJ2024029* and *CYJ2024032*). The descriptions of inflorescence are mainly based on the specimen *BPG II 23238*, which provided the epitype (designated here) of the species.

#### ﻿ Neomicrocalamus prainii

Taxon classificationPlantaePoalesPoaceae

(Gamble) Keng f.

A973A58A-930F-530E-A02B-03EAD0222A63

Neomicrocalamus prainii (Gamble) Keng f., J. Bamboo Res. 2(2): 10 1983.
≡ Microcalamusprainii Gamble, J. Asiat. Soc. Bengal, Pt. 2, Nat. Hist. 59(2): 207, pl. 7. 1891 (1890).
≡ Arundinariaprainii (Gamble) Gamble, Ann. Roy. Bot. Gard. (Calcutta) 7: 21 1896.
≡ Thamnocalamusprainii (Gamble) E.G. Camus, Bambusées: 54 1913.
≡ Racemobambosprainii (Gamble) Keng f. & T.H.Wen, J. Bamboo Res. 5(2): 13 1986.
= Melocalamusgracilis W. T. Lin, J. South China Agr. Univ. 14(3): 100, 1993. nom. illeg. [Its later homonym of Melocalamusgracilis R.B. Majumdar in S. Karthikeyan et al. Fl. Ind. ser. 4, 1 (Monocotyledon): 278. 1989. Type: INDIA • Barail Range, near Kailana, 9 km from Gumrirest house on Shillong, Cachar Road near P.W.D. Shed; R.B. Majumdar 1138 (holotype, CAL, not seen)] ≡ Melocalamusningmingensis Ohrnb. in D. Ohrnberger Bamb. World Introd. ed. 4: 19, 1997, syn. nov.

##### Type.

India • Assam: Naga Hills, 23 Apr 1886, alt. 2400 m, Dr. D. Prain s.n. (holotype, K!, Barcodes: K000246157; isotypes, K!, Barcodes: K000872509, K000872510, K000872511, K000872512; isotype, BM!, Barcode: BM000959211). China • Guangxi: Chongzuo City, Ningming County, Longrui, 1 Nov 1985, *Feipeng Chen 4726* (holotype, CANT!, Barcode: 25005).

##### Note.

*Melocalamusningmingensis* has a complex taxonomic history. Combining the Skmer analysis and morphological comparisons, we conclude that Feipeng Chen 4726 belongs to *Neomicrocalamusprainii* rather than *Melocalamus*.

#### ﻿ Melocalamus guangxiensis

Taxon classificationPlantaePoalesPoaceae

D.Z. Li & J.X. Liu sp. nov.

E83F5234-C685-5CE8-834E-47009D821531

urn:lsid:ipni.org:names:77364890-1

**Figure 7.**
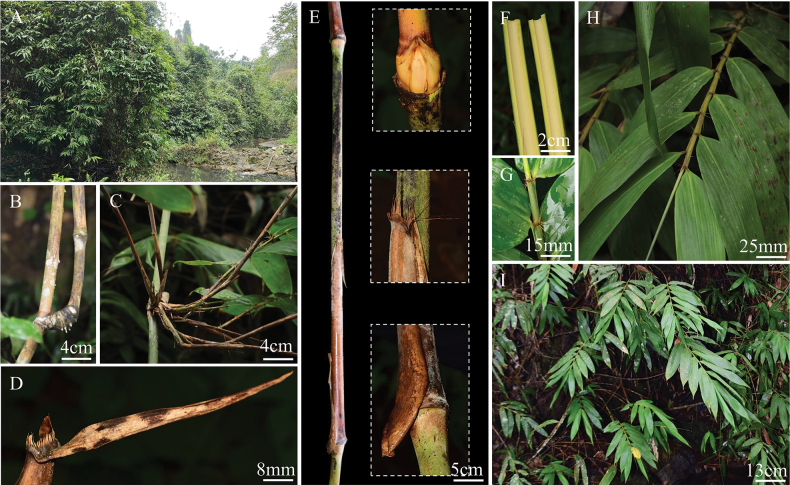
*Melocalamusguangxiensis* D.Z. Li & J.X. Liu **A.** Habitat; **B, C.** Branch complement; **D.** Culm leaf blade; **E.** A complete internode and culm leaf, showing the culm bud, apex of culm leaf sheath and the base of culm leaf sheath; **F.** Longitudinal section of main culm; **G, H.** Foliage leaves and foliage leaf sheath; **I.** Branchlet.

Fig. 7 Chinese name. “广西梨籐竹” (ɡuǎnɡ xī lí téng zhú).

##### Type.

China • Guangxi: Chongzuo City, Ningming County, Chengzhong Town, Shanghe Village, 22°11'54.10"N, 106°58'41.96"E, alt. 163 m, 11 Apr 2023, *Z.C. Xu et al. Xuzc2023109* (holotype, KUN!, Barcode: 1643898; isotype, CSH!, Barcode: CSH0219482).

##### Diagnosis.

*Melocalamusguangxiensis* resembles *M.puberulus*, *M.cordatus*, *M.pacoensis*, *M.truongsonensis* and M.compactiflorusvar.fimbriatus in the characters of foliage leaf auricles with radiate oral setae or culm leaf ligules with fringed long setae at the apex. However, *M.guangxiensis* can be easily distinguished by its unique culm sheaths, which feature a membranous projection on the basal outer margin, ca. 1–2 cm and culm leaf blades that are long- lanceolate, non-constricted at the base.

##### Description.

Perennial. Rhizomes pachymorph, short-necked. Culms slender, ca. 10–15 m long, 1–2 cm in diam.; internodes greyish-green, with densely white-brown pubescence, hollow, 50–80 cm long, wall 3–5 mm; nodes prominent, a ring of light-brown tomenta present above and below nodes; sheath scars prominent, corky, with residual base of culm leaf sheaths. Buds solitary, triangle, puberulent, the lateral edges ciliolate. Branches several, slender and equal length, dominant one equal to culm in size. Culm leaves tardily deciduous, ca. 2/5 to 1/2 as long as the internodes, 20–30 cm long, base 15–20 cm wide; sheaths leathery, long-triangle, apex concave or truncated, margins thin, membranous, with white powder and brown hairs abaxially, base of outer one with a subcircular projection, ca. 1–2 cm; auricles wavy, narrow rim, with several fimbriate long oral setae, easily dropped; ligules prominent, 2–3 mm in length, uniformly serrated, with fimbriate hairs, 7–14 mm long; blades length 6–8 cm × width 0.7–0.9 cm, lanceolate, erect or recurved. Foliage leaves 6–12 per ultimate branch; sheaths leathery, glabrous; auricles reflexed, sickle-shaped, oral setae radiated, 6–12 mm long; ligules ca. 3 mm in length, with white short hairs abaxially, apex truncate or with inconspicuous serrated, with fimbriate long hairs, ca. 1 cm long; blades lanceolate, 16–26 cm × 3–4.5 cm, with white pubescence abaxially, one margin entire, the other with short cilium, secondary veins 10–12 paired. Inflorescence and caryopsis unknown.

##### Phenology.

New shoots May to August.

##### Distribution and ecology.

This new species is found in Pingxiang and Congzuo Counties in Guangxi, China. It grows in warm and humid environments and usually occurs in limestone montane areas at altitudes of 100–300 m.

##### Etymology.

The epithet originates from Guangxi Autonomous Region where the new species was collected.

##### Additional specimen examined.

China • Guangxi: Chongzuo City, Pingxiang County, Xiashi Town, Pochatun, 22°11'7.14"N, 106°58'3.98"E, alt. 260 m, 21 Jul 2023, *C. Zhang et al. PX001*.

##### Note.

In the Skmer tree, sampled individuals of *Melocalamusguangxiensis* clustered in a clade sister to *M.putaoensis*. Due to notable morphological differences between *M.putaoensis* and *M.guangxiensis*, such as the absence of culm leaf auricles and oral setae, ligules without cilia and the base of outer margin in culm leaf sheaths without a membranous projection in *M.putaoensis*, we conclude that this species belongs to *Melocalamus*, but does not fit into any described species. Therefore, we treat it as a new species of *Melocalamus*.

### ﻿Remaining taxonomic problems

*Melocalamusfimbriatus* Hsueh & C.M. Hui was first described in 1992 (Hui and Hsueh 1992) and was subsequently treated as a variety of *Melocalamuscompactiflorus* in 2001 (Guo and Li 2001). Our phylogenetic result aligns with the topology constructed by Liu et al. (2023) using ddRAD-seq datasets, demonstrating that Melocalamuscompactiflorusvar.fimbriatus does not cluster within the same clade with *M.compactiflorus*, thereby suggesting a relatively distant genetic relationship between these two taxa. Furthermore, besides the differences noted by Guo and Li (2001) in the length and shape of the culm leaf ligules between these two taxa, we have also observed significant differences in their culm leaf auricles and other morphological characters. Currently, due to material limitations, only a single specimen of *M.compactiflorus* has been included in our Skmer analysis, thus, the phylogenetic position of this species needs further validation through expanded sampling efforts involving additional individuals. Subsequently, we will conduct further research to confirm the taxonomic status of M.compactiflorusvar.fimbriatus.

*Maclurochloa* K.M. Wong (Wong 1993) represents one of the bamboo genera distributed in Southeast Asia that exhibit climbing habits and possess a prominent branch. This genus differs from both *Melocalamus* and *Dinochloa* mainly in caryopsis type. Currently, four species have been formally described within this genus: *Maclurochloalocbacensis* H.N. Nguyen & V.T. Tran (Nguyen and Tran 2014), *Maclurochloamontana* (Ridl.) K.M. Wong (Wong 1993), *Maclurochloatonkinensis* H.N. Nguyen & V.T. Tran (Nguyen and Tran 2013) and *Maclurochloatrangdinhensis* H.N. Nguyen & V.T. Tran (Tran and Nguyen 2019). Upon reviewing relevant literature, we found that *Maclurochloa* shares high morphological similarities with certain *Melocalamus* species, most notably characterised by nodes covered with white powder and lanceolate pseudospikelets, gradually pointed at the apex. Moreover, we observed substantial morphological similarity between *Maclurochloatrangdinhensis* and *Melocalamusscandens*, both of them possessing culm leaf blades that are either equal in length or longer than the culm leaf sheaths and the junction between the blades as well as the sheaths being oblique. In addition, preliminary molecular studies implied that *Maclurochloamontana* fell into the BDG complex (Goh et al. 2010; Zhou et al. 2017), but the relationships with *Melocalamus* and *Soejatmia* K.M. Wong remain to be resolved. It is necessary to conduct more in-depth study on the phylogenetic relationship between the two genera, based on morphological and molecular data.

## Supplementary Material

XML Treatment for Cephalostachyum elevatissimum

XML Treatment for Melocalamus orenudus

XML Treatment for Neomicrocalamus prainii

XML Treatment for Melocalamus guangxiensis

## ﻿Additional information

### Author ORCIDs

Yu-Jin Chen https://orcid.org/0009-0003-3191-5078

Mei Chen https://orcid.org/0009-0004-0802-3669

Meng-Yuan Zhou https://orcid.org/0000-0003-1492-8494

Zu-Chang Xu https://orcid.org/0000-0001-7431-1061

Yu-Xiao Zhang https://orcid.org/0000-0003-0602-3118

Jing-Xia Liu https://orcid.org/0000-0003-4205-6400

De-Zhu Li https://orcid.org/0000-0002-4990-724X

### Data availability

All of the data that support the findings of this study are available in the main text or Supplementary Information.

## Supplementary materials

Supplementary material 1 Additional information Data type xlsx Explanation note **table S1.** Voucher information and sources of samples. **table S2.** The sequencing quality of samples. https://binary.pensoft.net/file/1366372This dataset is made available under the Open Database License (http://opendatacommons.org/licenses/odbl/1.0/). The Open Database License (ODbL) is a license agreement intended to allow users to freely share, modify, and use this Dataset while maintaining this same freedom for others, provided that the original source and author(s) are credited.Yu-Jin Chen, Mei Chen, Meng-Yuan Zhou, Zu-Chang Xu, Yu-Xiao Zhang, Jing-Xia Liu, De-Zhu Li

Supplementary material 2 The tree topology of the sampled *Melocalamus* with its related groups, based on the Skmer analysis Data type jpg Explanation note The tree topology of the sampled *Melocalamus* with its related groups, based on the Skmer analysis (without plastid and mitochondrial reads). Coloured boxes indicate the doubtful species and the species names in parentheses indicate the corresponding names before being revised in this study. Types of generic names are in bold. https://binary.pensoft.net/file/1366373This dataset is made available under the Open Database License (http://opendatacommons.org/licenses/odbl/1.0/). The Open Database License (ODbL) is a license agreement intended to allow users to freely share, modify, and use this Dataset while maintaining this same freedom for others, provided that the original source and author(s) are credited.Yu-Jin Chen, Mei Chen, Meng-Yuan Zhou, Zu-Chang Xu, Yu-Xiao Zhang, Jing-Xia Liu, De-Zhu Li
